# Supplementary material for: HIVprotI: an integrated web based platform for prediction and design of HIV proteins inhibitors
Source: J Cheminform. 2018 Mar 9;10:12. doi: 10.1186/s13321-018-0266-y (PMC5845081; doi:10.1186/s13321-018-0266-y)
Supplement: Supplementary file 2 — Additional file 2. Source code of HIVProtI web server. [file 13321_2018_266_MOESM2_ESM.zip › HIVprotI_Source-code/hivprotI/weka-3-6-6/doc/weka/experiment/xml/XMLExperiment.html]

XMLExperiment


---


|  |  |  |
| --- | --- | --- |
| **Overview** \| **Package** \| **Class** \| **Tree** \| **Deprecated** \| **Index** \| **Help** \| **Weka's home** | |  |
| PREV CLASS   NEXT CLASS | **FRAMES**    **NO FRAMES**     **All Classes** |  |
| SUMMARY: NESTED \| FIELD \| CONSTR \| METHOD | DETAIL: FIELD \| CONSTR \| METHOD |  |


---


## weka.experiment.xml Class XMLExperiment

```
java.lang.Object
  weka.core.xml.XMLSerialization
      weka.core.xml.XMLBasicSerialization
          weka.experiment.xml.XMLExperiment
```

**All Implemented Interfaces:**: RevisionHandler

---

``` public class XMLExperiment extends XMLBasicSerialization ```

This class serializes and deserializes an Experiment instance to and
fro XML.  
It omits the `options` from the Experiment, since these are handled
by the get/set-methods. For the `Classifier` class with all its
derivative classes it stores only `debug` and `options`.
For `SplitEvaluator` and `ResultProducer` only the
options are retrieved. The `PropertyNode` is done manually since
it has no get/set-methods for its public fields.  
Since there's no read-method for `m_ClassFirst` we always save it
as `false`.

**Version:**
:   $Revision: 1.6 $

**Author:**
:   FracPete (fracpete at waikato dot ac dot nz)

**See Also:**: `Experiment.m_ClassFirst`

---

| **Field Summary** | |
| --- | --- |
| `static java.lang.String` | `NAME_CLASSFIRST`             the name of the classFirst property |
| `static java.lang.String` | `NAME_PROPERTYNODE_PARENTCLASS`             PropertyNode member |
| `static java.lang.String` | `NAME_PROPERTYNODE_PROPERTY`             PropertyNode member |
| `static java.lang.String` | `NAME_PROPERTYNODE_VALUE`             PropertyNode member |

**Fields inherited from class weka.core.xml.XMLBasicSerialization**

`VAL_CELLS, VAL_KEY, VAL_MAPPING, VAL_VALUE`

**Fields inherited from class weka.core.xml.XMLSerialization**

`ATT_ARRAY, ATT_ARRAY_DEFAULT, ATT_CLASS, ATT_NAME, ATT_NULL, ATT_NULL_DEFAULT, ATT_PRIMITIVE, ATT_PRIMITIVE_DEFAULT, ATT_VERSION, DOCTYPE, ROOT_NODE, TAG_OBJECT, VAL_NO, VAL_ROOT, VAL_YES`


**Constructor Summary**

`XMLExperiment()`
  
          initializes the serialization


| **Method Summary** | |
| --- | --- |
| `void` | `clear()`             generates internally a new XML document and clears also the IgnoreList and the mappings for the Read/Write-Methods |
| `java.lang.String` | `getRevision()`             Returns the revision string. |
| `static void` | `main(java.lang.String[] args)`             for testing only. |
| `java.lang.Object` | `readPropertyNode(org.w3c.dom.Element node)`             builds the PropertyNode from the given DOM node. |
| `org.w3c.dom.Element` | `writePropertyNode(org.w3c.dom.Element parent, java.lang.Object o, java.lang.String name)`             adds the given PropertyNode to a DOM structure. |

**Methods inherited from class weka.core.xml.XMLBasicSerialization**

`readCollection, readCostMatrixOld, readDefaultListModel, readMap, readMatrix, readMatrixOld, writeCollection, writeCostMatrixOld, writeDefaultListModel, writeMap, writeMatrix, writeMatrixOld`

**Methods inherited from class weka.core.xml.XMLSerialization**

`fromXML, getVersion, read, read, read, read, readBooleanFromXML, readByteFromXML, readCharFromXML, readDoubleFromXML, readFloatFromXML, readFromXML, readFromXML, readIntFromXML, readLongFromXML, readShortFromXML, toXML, write, write, write, write, writeToXML`

**Methods inherited from class java.lang.Object**

`equals, getClass, hashCode, notify, notifyAll, toString, wait, wait, wait`

**Field Detail**

### NAME\_CLASSFIRST

```
public static final java.lang.String NAME_CLASSFIRST
```

:   the name of the classFirst property

    **See Also:**: Constant Field Values

---


### NAME\_PROPERTYNODE\_VALUE

```
public static final java.lang.String NAME_PROPERTYNODE_VALUE
```

:   PropertyNode member

    **See Also:**: Constant Field Values

---


### NAME\_PROPERTYNODE\_PARENTCLASS

```
public static final java.lang.String NAME_PROPERTYNODE_PARENTCLASS
```

:   PropertyNode member

    **See Also:**: Constant Field Values

---


### NAME\_PROPERTYNODE\_PROPERTY

```
public static final java.lang.String NAME_PROPERTYNODE_PROPERTY
```

:   PropertyNode member

    **See Also:**: Constant Field Values


**Constructor Detail**

### XMLExperiment

```
public XMLExperiment()
              throws java.lang.Exception
```

:   initializes the serialization

    **Throws:**: `java.lang.Exception` - if initialization fails


**Method Detail**

### clear

```
public void clear()
           throws java.lang.Exception
```

:   generates internally a new XML document and clears also the IgnoreList and
    the mappings for the Read/Write-Methods

    :   **Overrides:**: `clear` in class `XMLBasicSerialization`
    :   **Throws:**: `java.lang.Exception` - if initializing fails

---


### writePropertyNode

```
public org.w3c.dom.Element writePropertyNode(org.w3c.dom.Element parent,
                                             java.lang.Object o,
                                             java.lang.String name)
                                      throws java.lang.Exception
```

:   adds the given PropertyNode to a DOM structure.

    :   **Parameters:**: `parent` - the parent of this object, e.g. the class this object is a member of: `o` - the Object to describe in XML: `name` - the name of the object **Returns:**: the node that was created **Throws:**: `java.lang.Exception` - if the DOM creation fails

---


### readPropertyNode

```
public java.lang.Object readPropertyNode(org.w3c.dom.Element node)
                                  throws java.lang.Exception
```

:   builds the PropertyNode from the given DOM node.

    :   **Parameters:**: `node` - the associated XML node **Returns:**: the instance created from the XML description **Throws:**: `java.lang.Exception` - if instantiation fails **See Also:**: `DefaultListModel`

---


### getRevision

```
public java.lang.String getRevision()
```

:   Returns the revision string.

    :   **Specified by:**: `getRevision` in interface `RevisionHandler` **Overrides:**: `getRevision` in class `XMLBasicSerialization`
    :   **Returns:**: the revision

---


### main

```
public static void main(java.lang.String[] args)
                 throws java.lang.Exception
```

:   for testing only. if the first argument is a filename with ".xml"
    as extension it tries to generate an instance from the XML description
    and does a `toString()` of the generated object.
    Otherwise it loads the binary file, saves the XML representation in a
    file with the original filename appended by ".xml" and once again in a
    binary file with the original filename appended by ".exp".

    :   **Parameters:**: `args` - the commandline arguments **Throws:**: `java.lang.Exception` - if something goes wrong, e.g., file not found


---


|  |  |  |
| --- | --- | --- |
| **Overview** \| **Package** \| **Class** \| **Tree** \| **Deprecated** \| **Index** \| **Help** \| **Weka's home** | |  |
| PREV CLASS   NEXT CLASS | **FRAMES**    **NO FRAMES**     **All Classes** |  |
| SUMMARY: NESTED \| FIELD \| CONSTR \| METHOD | DETAIL: FIELD \| CONSTR \| METHOD |  |


---
